# CaMKII autophosphorylation is the only enzymatic event required for synaptic memory

**DOI:** 10.1073/pnas.2402783121

**Published:** 2024-06-18

**Authors:** Xiumin Chen, Qixu Cai, Jing Zhou, Samuel J. Pleasure, Howard Schulman, Mingjie Zhang, Roger A. Nicoll

**Affiliations:** ^a^Department of Neurology and Institute of Neuroscience of Soochow University, Second Affiliated Hospital of Soochow University, Suzhou 215004, China; ^b^Department of Cellular and Molecular Pharmacology, University of California, San Francisco, CA 94158; ^c^Division of Life Science, State Key Laboratory of Molecular Neuroscience, Hong Kong University of Science and Technology, Clear Water Bay, Kowloon, Hong Kong, China; ^d^Department of Laboratory Medicine, State Key Laboratory of Vaccines for Infectious Diseases, School of Public Heath, Xiamen University, Xiamen, Fujian 361102, China; ^e^Department of Neurology, University of California, San Francisco, CA 94158; ^f^Department of Pharmacology, Stanford University School of Medicine, Stanford, CA; ^g^Department of Pharmacology, Panorama Research Institute, Sunnyvale, CA; ^h^Department of Laboratory Medicine, School of Life Sciences, Southern University of Science and Technology, Shenzhen, Guangdong 518055, China

**Keywords:** CaMKII, CaMKII autophosphorylation, downstream kinase activity, synaptic memory, LTP

## Abstract

Ca^2+^/calmodulin (CaM)-dependent kinase II (CaMKII) plays a critical role in long-term potentiation (LTP), a well-established model for learning and memory through the enhancement of synaptic transmission. Biochemical studies indicate that CaMKII catalyzes a phosphotransferase (kinase) reaction of both itself (autophosphorylation) and of multiple downstream target proteins. However, whether either type of phosphorylation plays any role in the synaptic enhancing action of CaMKII remains hotly contested. We have designed a series of experiments to define the minimal requirements for the synaptic enhancement by CaMKII. We find that autophosphorylation of T286 and further binding of CaMKII to the GluN2B subunit are required both for initiating LTP and for its maintenance (synaptic memory). Once bound to the NMDA receptor, the synaptic action of CaMKII occurs in the absence of target protein phosphorylation. Thus, autophosphorylation and binding to the GluN2B subunit are the only two requirements for CaMKII in synaptic memory.

## Significance

The enzymatic action of Ca^2+^/calmodulin (CaM)-dependent kinase II (CaMKII) has two classes of targets. The first target class is itself, i.e., the autophosphorylation that maintains the enzyme in an open active conformation after the removal of Ca^2+^/CaM, providing a molecular memory. The second class of targets is the phosphorylation of multiple downstream synaptic proteins. While CaMKII is required for long-term potentiation (LTP), a compelling cellular model for learning and memory, the role of kinase activity in its synaptic actions is hotly debated. Here, we show that CaMKII autophosphorylation by promoting long-lasting binding to the GluN2B subunit of the N-methyl-D-aspartic acid receptor (NMDAR) is the only requirement for the maintenance of LTP. In addition, LTP is independent of the phosphorylation of downstream proteins.

The alpha isoform of Ca^2+^/calmodulin (CaM)-dependent kinase II enzyme (referred to throughout as CaMKII) has emerged as a central player in synaptic plasticity (1–7). While the activation of CaMKII is required for the induction of NMDA-dependent long-term potentiation (LTP), an established cellular model for learning and memory, the precise steps following its activation remains unclear. CaMKII is activated by an NMDA receptor-dependent rise in Ca^2+^ which together with CaM displaces an inhibitory segment of the kinase, converting the inactive closed conformation into an active open conformation. In the open conformation, CaMKII autophosphorylates T286 in the inhibitory segment, disables the inhibitory segment, and uncovers the binding sites for its substrates and anchoring proteins. The open conformation of CaMKII can be generated experimentally by use of the phosphomimic mutation T286D. Active (open) CaMKII binds (and phosphorylates) target substrate proteins as well as binding anchoring proteins such as the GluN2B subunit of the NMDAR.

The relative importance of T286 autophosphorylation, phosphorylation of downstream target proteins, and the binding to GluN2B remain hotly debated (5–7). Depending on the literature, one can select between two extreme scenarios: enzymatic and structural. In the enzymatic scenario, LTP is maintained by T286 autophosphorylation and the phosphorylation of target substrate proteins. In the structural scenario, LTP is maintained by the binding of CaMKII to GluN2B independent of any enzymatic activity, either of itself or toward target proteins.

To disambiguate among these scenarios, we determined the minimal requirements for the ability of CaMKII to enhance synaptic transmission and its role in the maintenance of LTP. We establish that T286 autophosphorylation is, indeed, required both for the induction and maintenance of LTP. Remarkably, a CaMKII kinase dead mutant (D135N), maintained in an open conformation by the additional T286D mutation, is fully capable of enhancing synaptic transmission. CaMKII synaptic function requires the binding to the GluN2B subunit of the NMDAR. We conclude that the only required kinase role of CaMKII in LTP is its autophosphorylation of T286, which, by enabling and stabilizing the CaMKII/GluN2B complex, maintains synaptic memory independent of phosphorylation of downstream targets.

After the completion of the present study and its appearance on BioRxiv (8), the study of Tulli et al. (9) appeared. This paper nicely complements our study. Although our experimental strategy is fundamentally different from theirs, we reached the same conclusion. Not addressed in their paper is whether autophosphorylation is required for the maintenance of synaptic memory. This issue has long been debated and we provide conclusive evidence that it is the autophosphorylation of CaMKII that sustains the memory.

## Results

### CaMKII-Mediated Synaptic Enhancement Is Independent of Substrate Protein Phosphorylation.

**Fig. 1.**
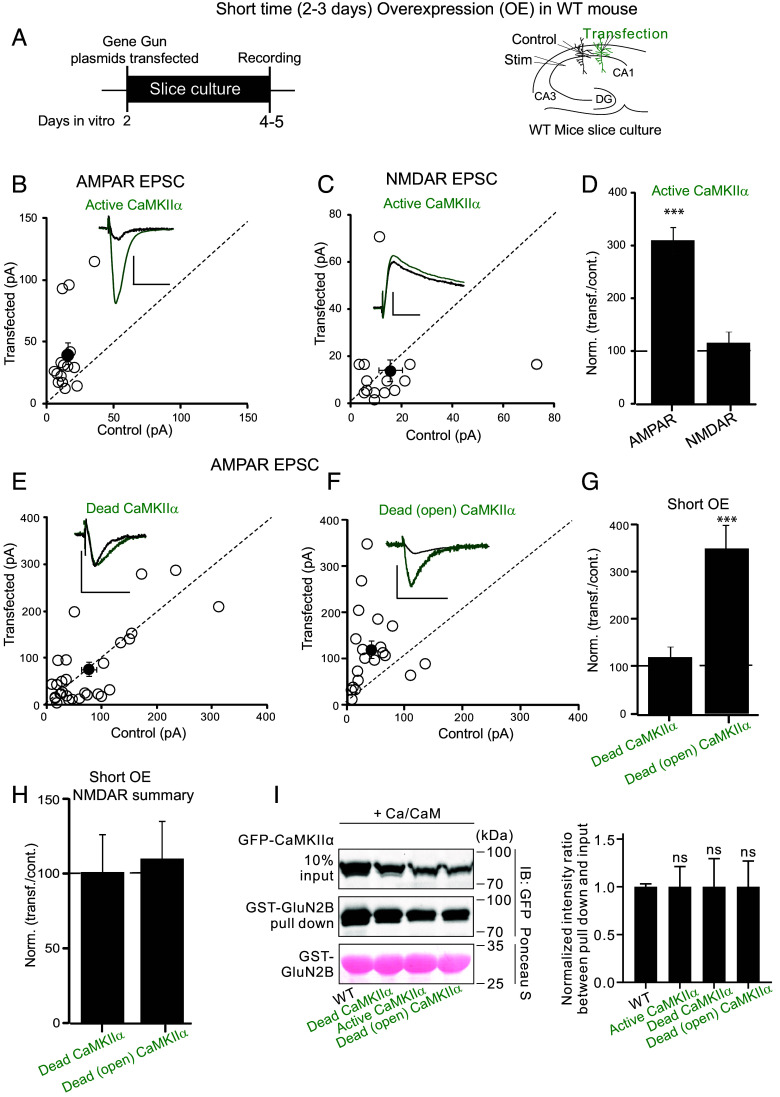
CaMKII-mediated synaptic enhancement is independent of substrate protein phosphorylation. (*A*) Schematic diagram showing the transfection, transfected constructs, and electrophysiological recording arrangement. Control represents the WT untransfected neurons. All experiments are from slice culture. (*B*) Scatterplots showing amplitudes of AMPAR EPSCs for single pairs (open circles) of control cell and cells overexpressing constitutively active CaMKII (T286D/T305A/T306A), referred to throughout as Active CaMKII for 2 to 3 d (short OE) (n = 15 pairs). Filled circle indicate mean ± SEM (Control = 15 ± 2; Active CaMKII short OE = 41 ± 8.8, *P* < 0.0001). (*C*) Scatterplots showing amplitudes of NMDAR EPSCs for single pairs (open circles) of control cells and cells transfected with Active CaMKII 2 to 3 d (short OE) (n = 15 pairs). Filled circles indicate mean ± SEM (Control = 15 ± 5; Active CaMKII short OE = 14 ± 5, *P* = 0.9). (*D*) Bar graph of ratios normalized to control (%) summarizing the mean ± SEM of AMPAR and NMDAR EPSCs of values represented in *B* (314 ± 26, *P* < 0.0001) and *C* (118 ± 20, *P* = 0.98). (*E*) Scatterplots showing amplitudes of AMPAR EPSCs for single pairs (open circles) of control and overexpressing cells of CaMKII D135N, referred to throughout as Dead CaMKII, 2 to 3 d (short OE) (*E*, n = 30 pairs). Filled circle indicate mean ± SEM (Control = 76.1 ± 13.9; Dead CaMKII short OE = 75.9 ± 15, *P* =0.98). (*F*) Dead CaMKII with the additional T286D/T305A/T306A mutation, referred to throughout as Dead (open) CaMKII 2 to 3 d (short OE) (*F*, n = 25 pairs). Filled circle indicate mean ± SEM [Control = 42.5 ± 8; Dead (open) CaMKII short OE = 120 ± 18.2, *P* < 0.0001]. (*G*) Bar graph of ratios normalized to control (%) summarizing the mean ± SEM of AMPAR EPSCs of values represented in *E* (120 ± 23, *P* = 0.6) and *F* (350 ± 50, *P* < 0.0001). (*H*) Bar graph of ratios normalized to control (%) summarizing the mean ± SEM of NMDAR EPSCs of Dead CaMKII short OE (101 ± 25, *P* = 0.6) and Dead (open) CaMKII short OE (95 ± 15, *P* = 0.7) Raw amplitude data from dual cell recordings were analyzed using Wilcoxon signed rank test (*P* values indicated above). (*I*) Representative GST GluN2B pull-down results using various mutants of CaMKIIα and bar graphs summarizing the results. Statistical data are presented as means ± SEM, with results from three independent batches of GST pull-down experiments. ns: not significant using one-way ANOVA with Dunnett’s multiple comparisons test. Normalized data were analyzed using a nonpaired *t* test followed by the Mann−Whitney test. (Scale bars, 30 ms, 50 pA.)

We first expressed a phosphomimic mutant of CaMKII, CaMKII (T286D/T305A/T306A) (10, 11), referred to throughout as Active CaMKII, which maintains the enzyme in an open conformation. We added the T305A, T306A mutations to obviate phosphorylation of T305 and T306 that block CaM binding to the open kinase and has other complicating effects (10). It is generally accepted that CaMKII is both necessary and sufficient for NMDAR-dependent LTP (7, 12, 13) and thus Active CaMKII is a useful surrogate for LTP. Simultaneous whole-cell recordings were made from a control cell and a transfected cell in wild-type (WT) slice culture and the responses from a common synaptic input compared (Fig. 1*A*). When overexpressed for a short period of time (2 to 3 d), referred to as short overexpression (short OE), Active CaMKII causes an approximately threefold enhancement in AMPAR responses (Fig. 1 *B* and *D*, n = 15), in general agreement with previous studies (10–17). Importantly, Active CaMKII has no effect on the NMDAR responses (Fig. 1 *C* and *D*, n = 15), thus mimicking LTP.

Does the enhancement require enzymatic activity? To answer this question, we used CaMKII (D135N), referred to throughout as Dead CaMKII, a mutation that blocks the catalytic activity (*SI Appendix*, Fig. S1*J*) by eliminating the catalytic base D135 without perturbing kinase structure (18). Importantly, this kinase dead mutation does not impact the binding to GluN2B in vitro (Fig. 1*I*), unlike the K42M kinase dead mutation (19). Short OE of Dead CaMKII on its own had no effect on AMPAR responses (Fig. 1*E*, n = 30). Remarkably, however, when this mutation was inserted into Active CaMKII, referred to as Dead (open) CaMKII, this kinase dead construct enhanced synaptic responses to the same degree as Active CaMKII when expressed either for short OE (Fig. 1 *F* and *G*, n = 25) or for 14 to 16 d (long OE) (*SI Appendix*, Fig. S1 *E–G*, n = 27). These findings are provocative because they indicate that phosphorylation of the numerous potential downstream targets is not necessary for the enhancing action of CaMKII.

Interestingly, the long overexpression (long OE) of Active CaMKII resulted in a substantial depression in both the AMPAR (*SI Appendix*, Fig. S1 *B* and *D*, n = 14) and the NMDAR responses (*SI Appendix*, Fig. S1 *C* and *D*, n = 14). In contrast, while long OE of Dead CaMKII depressed AMPAR responses (*SI Appendix*, Fig. S1 *E* and *G*, n = 25), the NMDAR responses were unaltered either with long OE of Dead CaMKII (*SI Appendix*, Fig. S1*H*, n = 25) or Dead (open) CaMKII (*SI Appendix*, Fig. S1*I*, n = 20). These findings showing that prolonged exposure of the synapse to Active CaMKII, but not to Dead CaMKII, has a deleterious effect on the synapse indicate that long-term phosphorylation of synaptic substrate proteins has a profound detrimental effect on synaptic function.

**Fig. 2.**
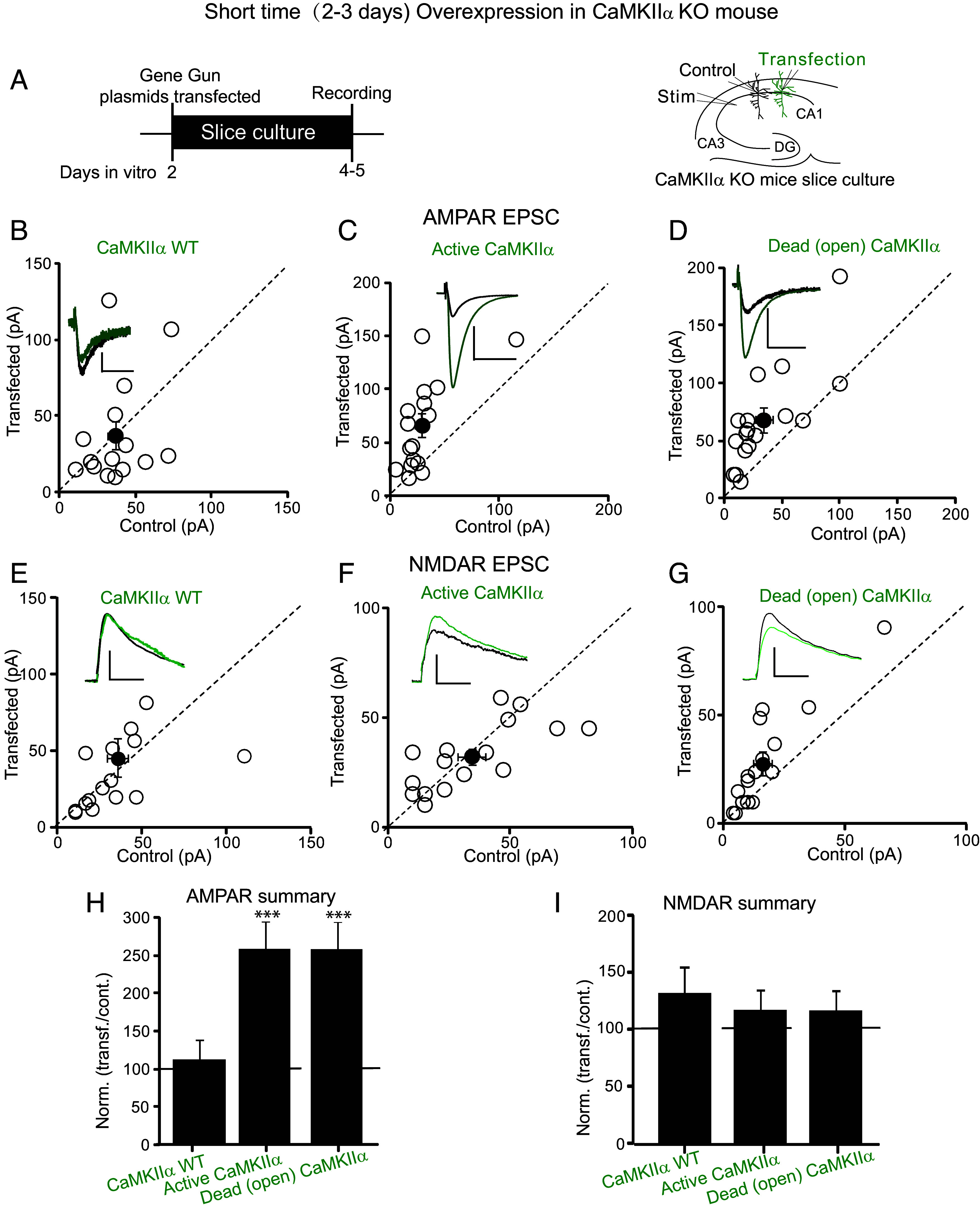
CaMKII-mediated synaptic enhancement is independent of substrate protein phosphorylation even in the absence of endogenous CaMKIIα. (*A*) Schematic diagram showing the transfection, transfected constructs, and electrophysiological recording arrangement. Control represents the untransfected neurons. All experiments are from slice culture prepared from CaMKIIα KO mice. (*B*–*D*) Scatterplots showing amplitudes of AMPAR EPSCs for single pairs (open circles) of control cells and cells expressing CaMKII WT for 2 to 3 d (short OE) (*A*, n = 20 pairs), Active CaMKII for 2 to 3 d (short OE) (*C*, n = 25 pairs), and Dead (open) CaMKII for 2 to 3 d (short OE) (*D*, n = 22 pairs) all in CaMKIIα KO mice slices. Filled circles indicate mean ± SEM [*B*, Control = 36.3 ± 4.7; CaMKII WT = 37.1 ± 9, *P* = 0.9; *C*, Control = 29.4 ± 6.1; Active CaMKII = 66.1 ± 10.6, *P* < 0.001; *D*, Control = 34.1 ± 7.5; Dead (open) CaMKII = 68.2 ± 10.7, *P* < 0.001]. (*E*–*G*) Scatterplots showing amplitudes of NMDAR EPSCs for single pairs (open circles) of control cells and cells expressing CaMKII WT for 2 to 3 d (short OE) (*E*, n = 20 pairs), Active CaMKII for 2 to 3 d (short OE) (*F*, n = 25 pairs), and Dead (open) CaMKII for 2 to 3 d (short OE) (*G*, n = 22 pairs) all in CaMKIIα KO mice slices. Filled circles indicate mean ± SEM [*E*, Control = 35.4 ± 6.4; CaMKII wt = 45.2 ± 12.5, *P* = 0.6; *F*, Control = 34.3 ± 5.5; Active CaMKII = 32.1 ± 3.8, *P* = 0.9; *G*, Control = 16.3 ± 3.7; Dead (open) CaMKII = 27.6 ± 5.7, *P* = 0.3]. (*H*) Bar graph of ratios normalized to control (%) summarizing the mean ± SEM of AMPAR EPSCs of values represented in *B* (113 ± 25, *P* = 0.8); *C* (263 ± 37, *P* < 0.001), and *D* (257 ± 33, *P* < 0.001). (*I*) Bar graph of ratios normalized to control (%) summarizing the mean ± SEM of NMDAR EPSCs of values represented in *E* (130 ± 23, *P* = 0.1); *F* (117 ± 18, *P* = 0.9), and *G* (121 ± 19, *P* = 0.2). Raw amplitude data from dual cell recordings were analyzed using Wilcoxon signed rank test (*P* values indicated above). Normalized data were analyzed using a one-way ANOVA followed by the Brown–Forsythe test and Bartlett's test. (Scale bars, 30 ms, 50 pA.)

The results with the Dead (open) CaMKII mutation suggest that the enhancing action of CaMKII does not require any downstream phosphorylation event. However, it is conceivable that the expressed Dead (open) CaMKII could exchange either by subunit exchange (20) or interholoenzyme phosphorylation (21) with the active endogenous CaMKIIαknown to be present at synapses (11, 22–24). To test this possibility, we made slices from the CaMKIIα KO mouse (Fig. 2*A*). Overexpression of WT CaMKII had no effect (Fig. 2 *B* and *H*, n = 20). This lack of effect is to be expected since there is no endogenous active CaMKIIα to exchange with the expressed WT CaMKII. On the other hand, overexpression of Active CaMKII (Fig. 2 *C* and *H*, n = 25) and Dead (open) CaMKII (Fig. 2 *D* and *H*, n = 22) still enhances synaptic transmission by threefold, confirming the conclusion that phosphorylation is not required for the enhancing action of expressed Active CaMKII. No change in the NMDAR response was observed with any of these manipulations (Fig. 2 *E*–*G* and *I*). One might argue that the presence of endogenous CaMKIIβ in these experiments provides another source for phosphorylation. Importantly, however, when the long OE experiments of Dead (open) CaMKII (*SI Appendix*, Fig. S1 *E–G*) were carried out on the CaMKIIα/CaMKIIβ null background (DKO CaMKII), the enhancing action of Dead (open) CaMKII remained intact, confirming the kinase independence of the enhancement.

### CaMKII Autophosphorylation and Binding to GluN2B Are Necessary for Its Synaptic Action.

**Fig. 3.**
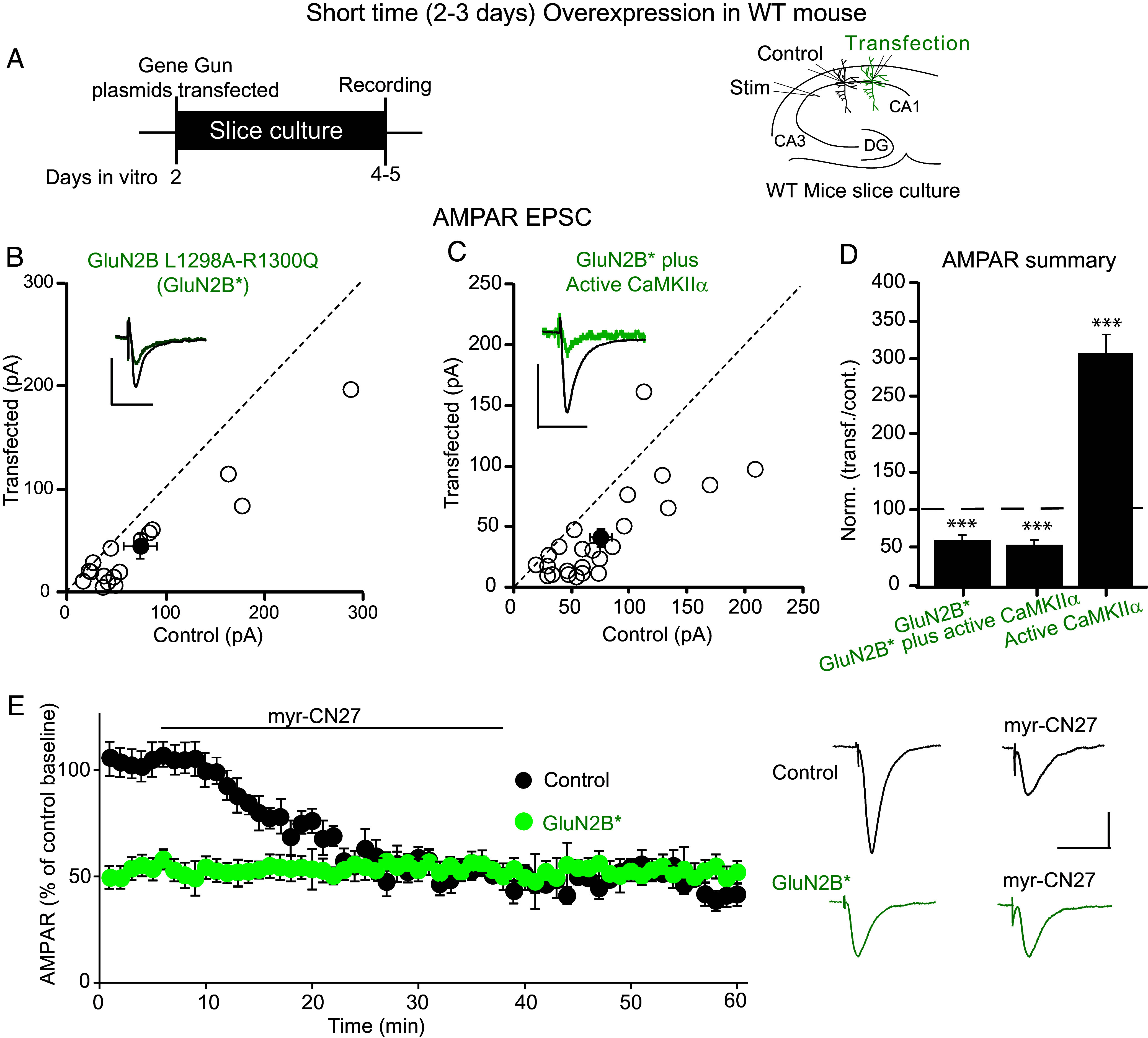
CaMKII binding to GluN2B is necessary for its synaptic action. (*A*) Schematic diagram showing the transfection, transfected constructs, and electrophysiological recording arrangement. Control represents the WT untransfected neurons. All experiments are from slice culture. (*B* and *C*) Scatterplots showing amplitudes of AMPAR EPSCs for single pairs (open circles) of control cells and cells expressing GluN2B*(GluN2B L1298A-R1300Q) 2 to 3 d (short OE) (*B*, n = 20 pairs) and GluN2B*+ Active CaMKII 2 to 3 d (short OE) (*C*, n =31 pairs). Filled circles indicate mean ± SEM [*B*, Control = 73.4 ± 16.8; GluN2B*(GluN2B L1298A-R1300Q) short OE = 44.9 ± 11.6, *P* < 0.01; *C*, Control = 74.9 ± 9.4; GluN2B*+ CA CaMKII short OE = 41.5 ± 7.4, *P* < 0.01]. (*D*) Bar graph of ratios normalized to control (%) summarizing the mean± SEM of AMPAR EPSCs (61.5 ± 7, *P* < 0.001). OE CA CaMKIIα 2 to 3 d data from Fig. 1*D* were included in the graph. (*E*) Plots show mean ± SEM AMPAR EPSC amplitude of control cells (filled circles, n = 12) and cells transfected with GluN2B* (green circles, n = 5, three simultaneous recordings included). Raw amplitude data from dual cell recordings were analyzed using Wilcoxon signed rank test (*P* values indicated above). Normalized data were analyzed using a one-way ANOVA followed by the Brown–Forsythe test and Bartlett’s test. (Scale bars, 30 ms, 50 pA.)

Biochemical studies indicate that CaMKII binds to GluN2B (19, 25–28), and disrupting this binding impairs LTP (29, 30). To directly address the importance of this CaMKII-GluN2B binding for the synaptic action of CaMKII, we overexpressed Active CaMKII under conditions that prevent the binding of Active CaMKII to GluN2B (Fig. 3*A*). Overexpressing a mutant form of GluN2B (L1298A-R1300Q; GluN2B*), in which binding to the CaMKII surface groove is disabled (29), on a WT background causes a ~50% depression in AMPAR responses after short OE (Fig. 3 *B* and *D*, n =20). Might this depression be due to the disruption of the CaMKII action that maintains basal synaptic transmission (11, 22–24)? To test this possibility, we turned to myr-CN27, which selectively blocks the action of CaMKII (22). We recorded simultaneously from control cells or cells expressing GluN2B* (Fig. 3*E*). The size of the AMPAR EPSC in cells expressing GluN2B* (green circles) was approximately 50% of that of the control cells (filled circles). While myr-CN27 had its usual ~50% depression in control cells, it had no effect on simultaneously recorded cells expressing GluN2B* (Fig. 3*E*), indicating that the binding of CaMKII to GluN2B is necessary for the constitutive action of CaMKII on basal synaptic transmission. These findings predict that the expression of Active CaMKII in the presence of GluN2B* should no longer enhance synaptic responses. Indeed, the short OE of GluN2B*+Active CaMKII generates responses no larger than those recorded with GluN2B* alone (Fig. 3 *C* and *D*, n =31). As a control, we expressed WT GluN2B together with Active CaMKII (*SI Appendix*, Fig. S2 *B–D*). The expression of WT GluN2B had no effect on the enhancing action of expressing Active CaMKII on its own (*SI Appendix*, Fig. S2 *B* and *D*). Furthermore, expression of WT GluN2B together with Dead (open) CaMKII (*SI Appendix*, Fig. S2 *E–G*) had no effect on the enhancing action of expressing Dead (open) CaMKII. There was no change in the NMDAR responses to either manipulation (*SI Appendix*, Fig. S2 *C* & *D* and *F* & *G*) Thus, these results indicate that CaMKII binding to GluN2B is required both for the constitutive action of CaMKII in maintaining synaptic responses as well as in the enhancing action of Active CaMKII.

### CaMKII Autophosphorylation Is Required for LTP Induction.

**Fig. 4.**
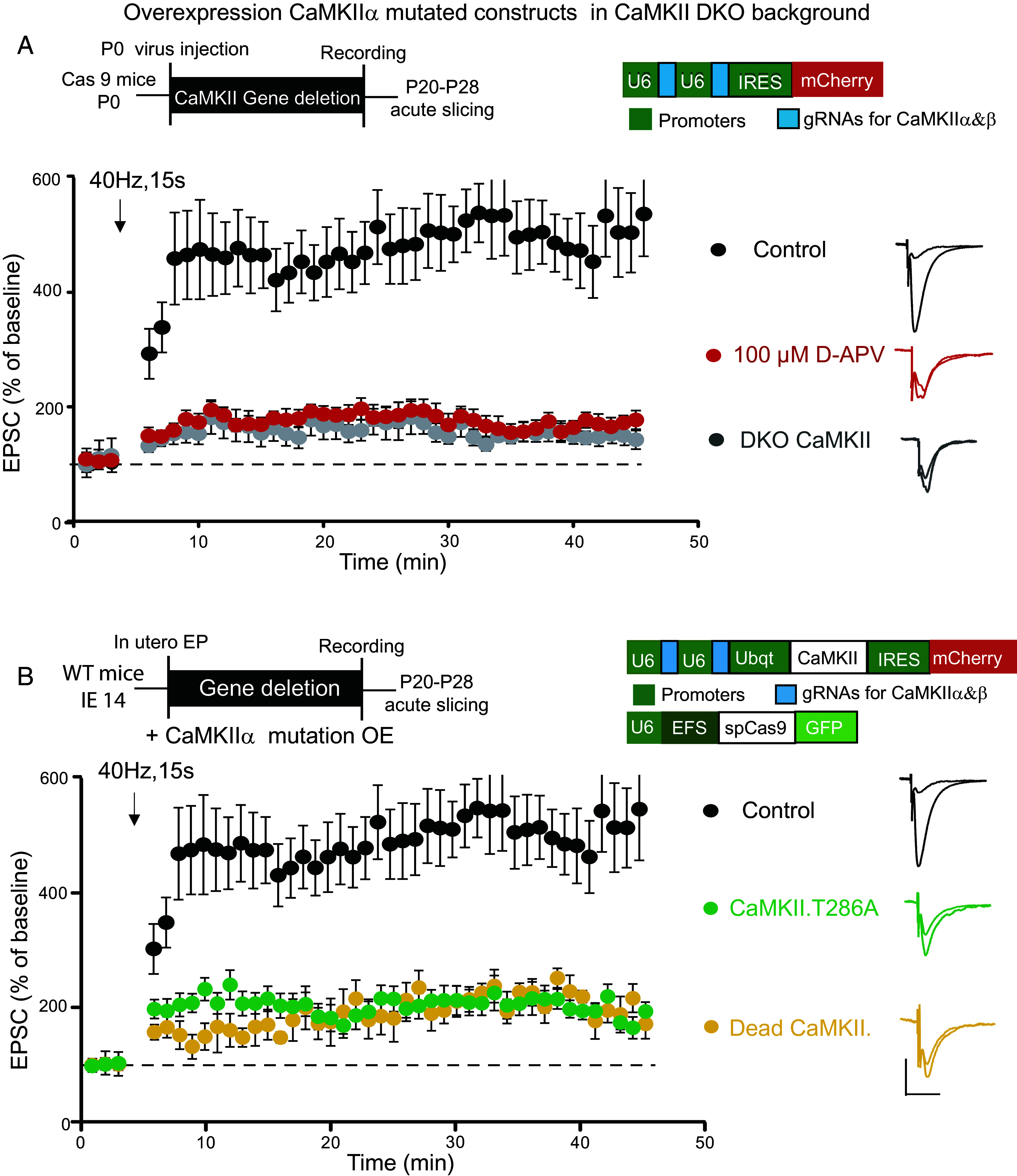
CaMKII T286A and Dead CaMKII fail to support LTP. (*A*) Schematic diagram showing the transfection approach and gRNA construct which were used only for the CRISPR DKO experiment. All experiments are from acute slices. 40 Hz, 15 s pairing protocol generates robust LTP in WT CA1 hippocampal cells (n = 19 control cells, filled circles). LTP is largely blocked by 100 μM D-APV (red circles, n = 8) and CRISPR DKO CaMKII (CaMKIIα and CaMKIIβ) (gray circles, n = 10, six simultaneous recordings included). (*B*) Schematic diagram showing the transfection approach and constructs. All experiments involved expressing either T286A or Dead CaMKII on a CaMKII null background. NMDAR-dependent LTP is absent in cells expressing Dead CaMKII (Yellow circles, n = 11, seven simultaneous recordings included) or CaMKII T286A (green circles, n = 10, six simultaneous recordings included). For all LTP graphs, control cells are shown as filled circles ±SEM and transfected cells are shown as colored circles ±SEM as defined. Traces show representative currents before and after LTP induction. (Scale bar, 50 pA/30 ms.)

Our results indicate that a kinase dead mutant of CaMKII held in an open configuration [Dead (open) CaMKII] is fully functional in potentiating synapses. The remaining question is whether autophosphorylation is required for the action of CaMKII. In theory, the action of CaMKII in LTP could be purely structural: The interaction of Ca^2+^/CaM with CaMKII, by promoting the open state conformation, would enable the binding of the kinase to GluN2B, without the need of any enzymatic activity. Indeed, a recent study (31) reported NMDAR-dependent LTP in the phosphonull mutant (T286A) knockin mouse. We therefore re-examined the role of phosphorylation in the induction of LTP using the pairing protocol of Chang et al. (31) (pairing 40 Hz, 15 s with 0 mV depolarization). With this protocol, we observe an LTP that is similar in magnitude to that observed with our standard induction protocol (pairing 2 Hz, 90 s with 0 mV depolarization), e.g., refs. 32 and 33 (Fig. 4*A*, filled circles). Note that pairing 40 Hz, 15 s with 0 mV depolarization protocol evokes a small residual LTP that remains in the presence of the NMDAR blocker APV (Fig. 4*A*, red circles, n = 8) and in cells lacking both CaMKIIα and CaMKIIβ (Fig. 4*A*, gray circles, n = 10). Such an NMDAR-independent form of LTP has been reported with a robust (200 Hz tetanus) induction protocol (34, 35). The present study is focused on the NMDAR/CaMKII-dependent form of LTP. We next replaced endogenous CaMKII with a kinase dead mutant (Dead CaMKII). However, no LTP was recorded (Fig. 4*B*, yellow circles, n = 11), in agreement with a previous study using the K42R kinase dead mutant knockin mouse (36). Finally, we examined LTP in cells expressing CaMKII (T286A) on a null background. We were unable to detect NMDAR-dependent LTP (Fig. 4*B*, green circles, n = 10). The only difference between these experiments and those of Chang et al. is that they used T286A knockin mice, while we used in utero electroporation. It is unclear whether this difference explains the different results.

### CaMKII T286 Autophosphorylation Is Required for Maintaining Synaptic Memory.

**Fig. 5.**
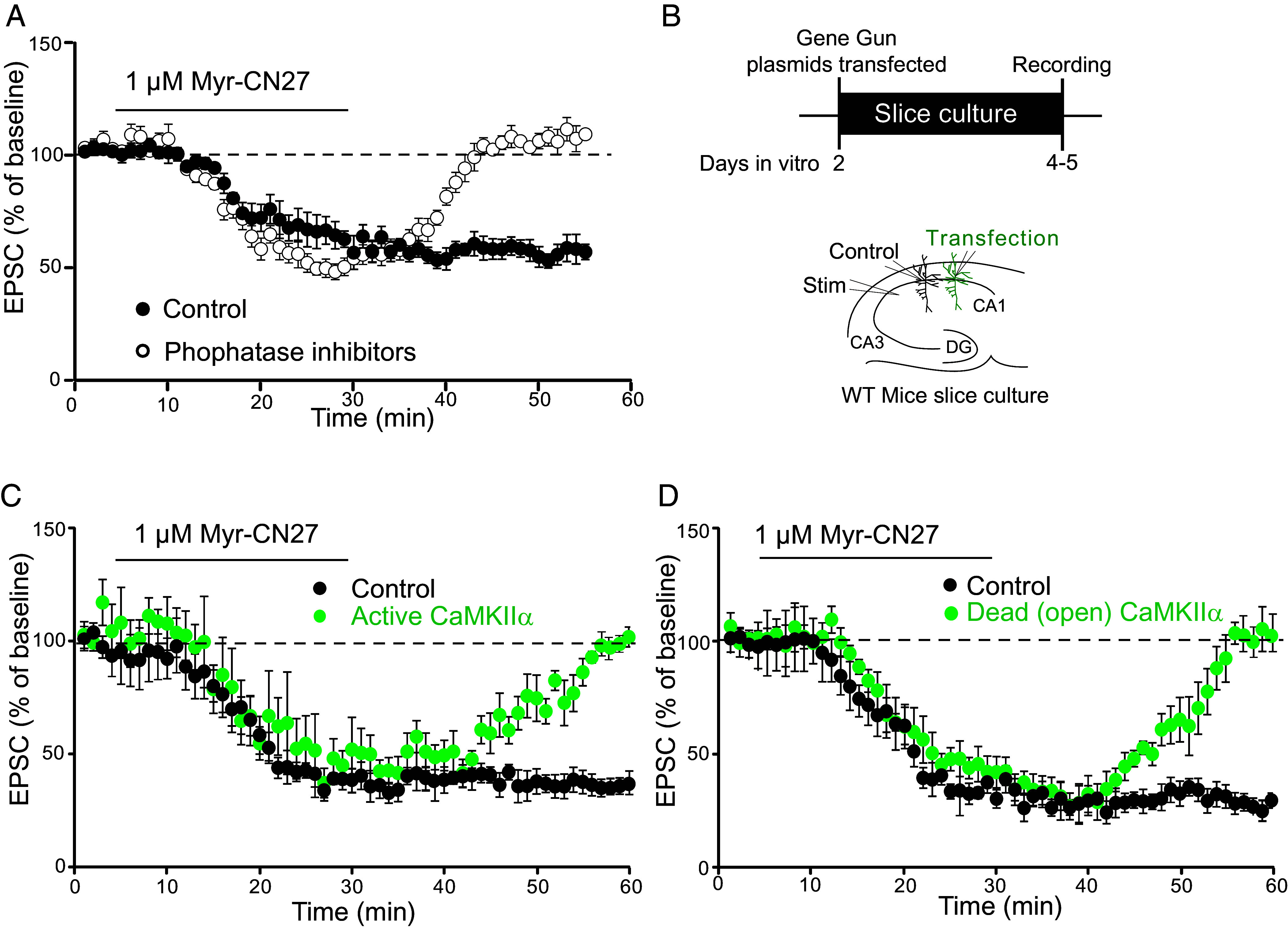
Only CaMKII T286 phosphorylation is required to maintain CaMKII synaptic memory. (*A*) Plot (filled circles) shows that transient application of 1 μM myr-CN27 (filled circles, n = 20) causes a long-lasting decrease in AMPAR EPSCs (±SEM). When this experiment is repeated in the presence of phosphatase inhibitors (open circles, n = 23), the AMPAR responses rapidly return to baseline. (*B*) shows the transfection approach and the electrophysiological recording arrangement, which applies to (*C* and *D*). (*C*) Plot shows mean AMPAR EPSC (±SEM) amplitude of control cells (filled circles, n = 8) and cells transfected with Active CaMKII (green circles, n = 12, five simultaneous recordings included). Note the recovery of the synaptic responses. (*D*) Plot shows mean AMPAR EPSC (±SEM) amplitude of control cells (filled circles, n = 13) and cells transfected with Dead (open) CaMKII (green circles, n = 7, six simultaneous recordings included). Note the rapid recovery of the synaptic responses, confirming that phosphorylation is not required for maintaining the synaptic action of CaMKII.

The results presented thus far indicate that T286 must be autophosphorylated for the induction of LTP by WT CaMKII. Is the autophosphorylation also required for the maintenance of LTP? There is a long history challenging the role of CaMKII enzymatic activity in LTP maintenance (23, 31, 37–41) To address this issue, we turned to the endogenous constitutive CaMKII component of synaptic transmission (11, 22–24). We (22) and others (23, 42) have presented evidence that this constitutive CaMKII action, either enzymatic or structural (i.e., binding to GluN2B), represents a CaMKII synaptic memory trace acquired prior to slice preparation. Does this trace require continued T286 autophosphorylation? The bath application of the CaMKII inhibitor myr-CN27 reverses this memory trace and importantly, there is no recovery of synaptic transmission following the wash out of the inhibitor (Fig. 5*A*, filled circles, n = 20) (22, 24). It is important to keep in mind that CN27 has two actions: it blocks kinase activity (43–46) and it disrupts the binding of CaMKII to GluN2B (18, 23, 46). Thus, the depression caused during the myr-CN27 application is likely due to disruption of the binding of CaMKII to GluN2B. The failure of the responses to recover after washout of myr-CN27 would indicate that the released CaMKII is incapable of rebinding to GluN2B, perhaps due to its dephosphorylation to a level below the threshold necessary for rebinding and constitutive activity. If this is the case, one would predict that repeating this experiment in the presence of phosphatase inhibitors should allow for the rebinding of the autophosphorylated CaMKII to GluN2B.

Phosphatase inhibitors were either bath applied (calyculin A, n =11) or added to the pipette solution (microcystin LR, n = 12). Since both manipulations gave the same result, we have combined the data. The inhibitors have no effect on the magnitude of the synaptic depression during the myr-CN27 application (Fig. 5*A*, open circles). However, following the washout of myr-CN27, the responses rapidly recover in the presence of phosphatase inhibitors (Fig. 5*A*, open circles). These findings suggest that the acute inhibition caused by myr-CN27 is due to the dissociation of CaMKII from GluN2B and not the dephosphorylation of T286. To test this idea further, we repeated the experiment in neurons expressing phosphatase-resistant forms of CaMKII (Fig. 5*B*). In neurons expressing either Active CaMKII (Fig. 5*C*, green circles, n = 7) or the Dead (open) CaMKII (Fig. 5*D*) green circles, n = 7), the responses rapidly recovered. In contrast, as expected, in either simultaneously recorded control cells (n = 15) or interleaved cells (n = 11), there was no recovery following the transient application of myr-CN27. Taken together, these results indicate that the depression in synaptic transmission during the application of myr-CN27 is due to the dissociation of active CaMKII from GluN2B, while the persistent depression following wash out of the inhibitor is due to the dephosphorylation of T286 and the failure of dephosphorylated CaMKII to rebind to GluN2B. The results with Dead (open) CaMKII further confirm that no phosphorylation is necessary for recovery. These results show that the maintenance of LTP requires T286 autophosphorylation and that binding of CaMKII to GluN2B shields this phosphorylation from phosphatases.

## Discussion

**Fig. 6.**
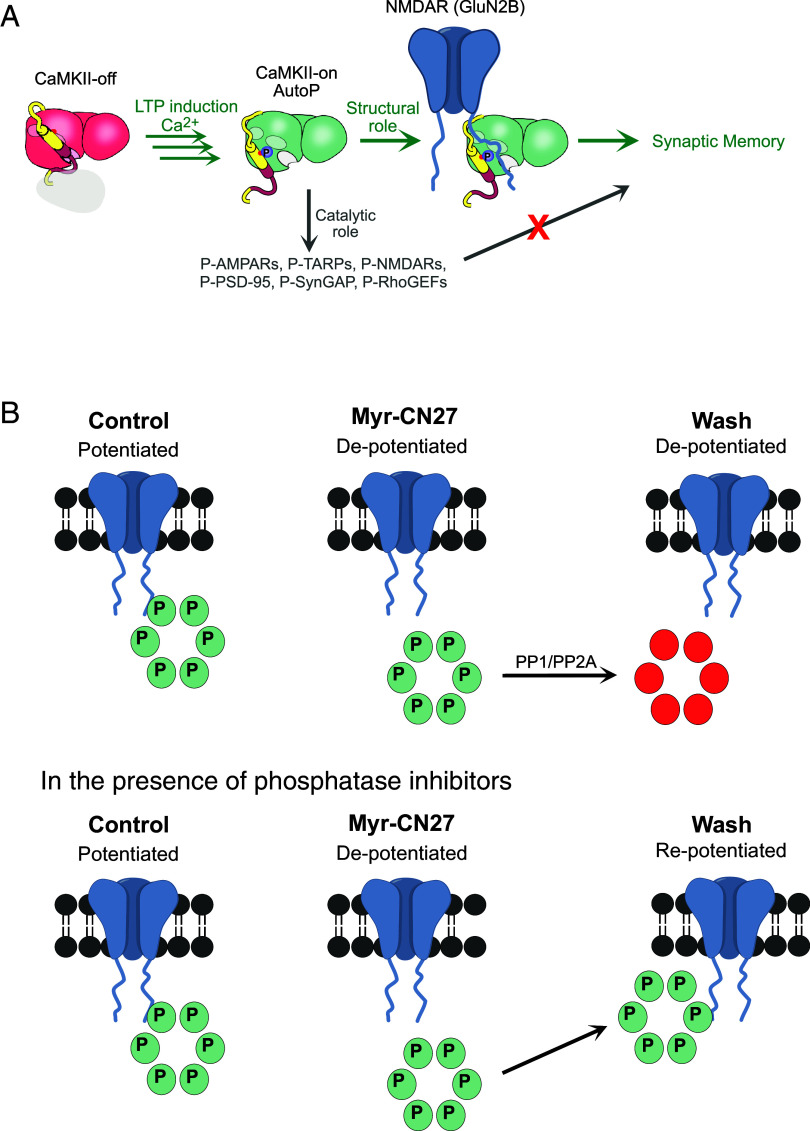
Autophosphorylation of CaMKII T286 is required for synaptic memory, but not phosphorylation of downstream synaptic proteins. (*A*) Autophosphorylation of CaMKII T286 is required for synaptic memory by stabilizing the binding of CaMKII to GluN2B. The numerous proposed downstream synaptic protein targets of CaMKII are not required for synaptic memory. Rather, the CaMKII/GluN2B complex serves as a structural signaling hub to enhance and maintain synaptic strength. (*B*) The stability of the CaMKII/GluN2B complex requires the continued phosphorylation of T286 to maintain the synaptic memory. Transient application of myr-CN27 (Fig. 5) dissociates active CaMKII from GluN2B. The released active CaMKII is rapidly dephosphorylated by phosphatases, erasing the synaptic memory. In the presence of phosphatase inhibitors, the active CaMKII rapidly rebinds to GluN2B restoring the synaptic memory.

Biochemical studies have established that CaMKII has two classes of targets of its enzymatic actions: autophosphorylation of CaMKII T286, which maintains the enzyme in an open active state, and phosphorylation of downstream targets, which has been proposed to enhance synaptic transmission. However, the role of these biochemical properties of CaMKII in LTP and the synaptic action of CaMKII remains confusing. The high levels of CaMKII in the PSD, ranging up to 10 to 30% (47–49) have long raised the possibility of a structural role for CaMKII (50, 51). We have used four different mutant forms of CaMKII to distinguish between the enzymatic and structural roles in its synaptic enhancing action and in LTP. Our findings lead to a number of conclusions. First, the phosphorylation of T286 is required for LTP and the synaptic action of CaMKII (Fig. 6*A*). Second, the binding of CaMKII to GluN2B is required for the action of CaMKII (Fig. 6*A*). Third, the phosphorylation of synaptic proteins is not required for the action of CaMKII (Fig. 6*A*). It is uncertain to what degree synaptic proteins involved in LTP are phosphorylated by CaMKII in vivo. Given that prolonged expression of Active CaMKII is profoundly deleterious to synaptic function, we propose that the sequestering of active CaMKII by GluN2B shields the synaptic proteins from phosphorylation. Fourth, the maintenance of synaptic potentiation requires persistent T286 phosphorylation and the binding of active CaMKII to GluN2B (Fig. 6*B*).

Our use of the term “synaptic memory” includes NMDAR-dependent LTP, the constitutive action of CaMKII and related phenomena, such as the formation and maintenance of hippocampal place fields, which is dependent on both NMDARs (52) and CaMKII (53). The changes occurring at individual synapses require further study. There is clearly a population of synaptic AMPARs that are independent of both NMDAR and CaMKII activation, since synaptic AMPAR EPSCs are present after the deletion of NMDARs (54) or CaMKII (11) prior to synaptogenesis. The mechanism of this NMDAR/CaMKII-independent AMPAR trafficking is unclear. This paper is concerned with the NMDAR and/or CaMKII-dependent AMPAR trafficking. Based on studies of synaptic plasticity at individual synapses (55–59), we would predict that the action of CaMKII at individual synapses is heterogeneous, including synapses that are insensitive, synapses with AMPARs that acquire additional AMPARs and synapses without AMPARs (silent synapses) that acquire a full complement of AMPARs. The relative contributions of these synaptic changes to behavioral plasticity remains to be established.

We confirm previous results (22, 24) showing that transient application of CN27 or AIP2, which disrupts the binding of CaMKII to GluN2B, results in a depression in synaptic transmission that long outlives the brief application (Fig. 6*B*). The lack of recovery is due to the rapid dephosphorylation of the released CaMKII by phosphatases, consistent with previous proposals that binding of CaMKII to GluN2B shields active CaMKII from phosphatases (3, 60–62). Most importantly, the lack of recovery of synaptic transmission following the transient application of myr-CN27 indicates that in our slice culture experiments, any spontaneous fluctuations in spine Ca^2+^ levels are insufficient for endogenous CaMKII exert its physiological effect.

Our findings emphasize the central role of the NMDAR in LTP; it initiates LTP by increasing spine Ca^2+^ levels and it maintains LTP by its binding of CaMKII. T286 autophosphorylation of CaMKII is required to stabilize the CaMKII/GluN2B complex. This finding nicely compliments the recent phase separation studies of Hosokawa et al. (63) and Cai et al. (64) showing that while the condensates formed by Ca^2+^/CaM-bound CaMKII and GluN2B do not require T286 phosphorylation, it is required for the condensates to remain intact following the removal of Ca^2+^/CaM.

It is remarkable that phosphorylation of the many identified downstream synaptic target proteins of CaMKII, such as GluA1, TARPs, synGAP, RhoGEFs, PSD-95, etc., that have long been implicated in LTP (reviewed in refs. 2, 7, and 65), is dispensable for its synaptic enhancement (Fig. 6*A*), thus establishing a structural role of CaMKII. In fact, the prolonged phosphorylation of one or more synaptic proteins by expression of Active CaMKII had a profound deleterious effect on both AMPAR and NMDAR responses. This finding implies that during the maintenance of LTP, the active CaMKII is prevented access to these target proteins by its sequestered binding to GluN2B. What role these target proteins play in the action of CaMKII requires further investigation. The present results focus attention on the CaMKII/GluN2B complex as a structural hub. Again, the recent studies of Hosokawa et al. (63) and Cai et al. (64) are informative. The authors show that CaMKII and GluN2B undergo phase separation forming lasting condensates after the removal of Ca^2+^/CaM. However, phosphatase can rapidly remove phosphate from T286 causing the rapid disassembly of CaMKII/GluN2B condensates. Our results showing that releasing active CaMKII from GluN2B results in its rapid dephosphorylation by phosphatases provides physiological validation for these phase separation results. Furthermore, the addition of PSD-95 and TARPs, a proxy for AMPARs, results in a distinct phase in phase separation (63). Except for T286 phosphorylation, this molecular assembly is independent of phosphorylation, again in accord with our physiological results. It will be of interest to establish whether the phase separation so clearly established with the synaptic component proteins in solution can also explain our present results with intact synapses.

Finally, our study reveals that the enzymatic activity of synaptic CaMKII is dedicated to the regulation of its open state via autophosphorylation, thereby allowing this extremely abundant enzyme to function as a synaptic scaffold protein. Since autophosphorylation and consequent conformational change is a rather general property of protein kinases, it will be interesting to explore how many other protein kinases also adopt similar autophosphorylation-regulated structural roles for cellular processes.

## Methods

### DNA Constructs and Chemical Agents.

The cDNA sequences of rat CaMKIIα (Uniprot: P11275) were PCR-amplified from rat brain cDNA library. Various mutants of CaMKII were generated by the standard PCR-based method and individually inserted into a pEGFP-C3 vector with N-terminal EGFP tag for expression in HEK293T cells for GST pull-down experiments. CaMKII DKO constructs have been described and validated previously (11). Briefly, CaMKII deletion was generated by coexpression of CaMKIIα and CaMKIIβ gRNA with Cas9 for over two weeks. All expression vectors for CaMKII KO and replacement were constructed within a lentiviral backbone FUGW. The FUGW construct including a Ubiquitin C promoter follows two gRNA sequences to drive CaMKII rescue constructs and an IRES promotor for mCherry expression. The four CaMKIIα mutate constructs used in this study include the following: Dead CaMKII (i.e., CaMKII (D135N), Active CaMKII (i.e., T286D+T305A+T306A), Dead (open) CaMKII (i.e., D135N+T286D+T305A+T306A), and CaMKII (T286A) were incorporated into the above FUWG vector. For the LTP experiments in Fig. 4*A*, the px458 vector and Rosa26-Cas9 knock-in mice were used for Cas9 expression (66). GluN2B mutation expression construct was located in pCAGGS plasmid followed by IRES and GFP, which was also described and validated previously (11). All mutants were generated by the standard PCR-based method. All constructs were confirmed by DNA sequencing.

Myr-CN27 (Calbiochem. Inc. catalog # 208921), microcystin LR (Sigma-Aldrich. catalog # 475823), and calyculin A (VWR. catalog # EI192) were purchased from the above sources.

### Lentivirus Production.

Three T-75 flasks of rapidly dividing HEK293T cells (ATCC) were transfected with 27 mg FUGW-CaMKII 2gRNA-CaMKII variant-mCherry, plus helper plasmids pVSV-G (18 mg) and psPAX2 (27 mg) using FuGENE HD (Promega). DNA was incubated with 210 mL FuGENE HD in 4.5 mL Opti-MEM (Life Technologies) before transfection, according to the manufacturer’s directions. After 40 h and then again at 3 d, the supernatant was collected, filtered, and concentrated using the PEG-it Virus Precipitation Solution (System Biosciences) according to the manufacturer’s directions. The resulting pellet was resuspended in 150 mL Opti-MEM, flash-frozen with dry ice, and stored at −80 °C.

### In Utero Electroporation.

In utero electroporation was performed as previously described (11). E14 pregnant WT (CD-1) mice were anesthetized with 2% isoflurane. Embryos were exposed and injected with ~1.5 μL of mixed plasmid DNA with Fast Green (Sigma Aldrich) into the lateral ventricle via a beveled micropipette. Each embryo was electroporated with five 27 V pulses of 50 ms, delivered at 1 Hz, using platinum tweezer-trodes in a square-wave pulse generator (BTX Harvard Apparatus). The positive electrode was placed in the lower right hemisphere and the negative electrode was placed in the upper left hemisphere. Following electroporation, the embryos were placed back into the abdominal cavity and abdominal muscle and skin were sutured. All experiments were performed in accordance with the established protocols approved by the University of California, San Francisco’s Institutional Animal Care and Use Committee.

### Acute Slice Preparation.

Acute hippocampal slices for the LTP experiments in Fig. 4*B* were prepared from P18 to P28 mice. Mice were anesthetized with isoflurane. Brains were removed and sliced into 300 µm near-horizontal sections using Microslicer DTK-Zero1 (Ted Pella). Slices were then transferred to a holding chamber containing ACSF (in mM) (125 NaCl, 2.5 KCl, 1.25 NaH_2_PO_4_, 25 NaHCO_3_, 11 glucose, 1 MgSO_4_, 2 CaCl_2_ saturated with 95% O_2_/5% CO_2_) and incubated for 20 min at 37 °C and then kept at room temperature until use.

### Slice Culture and Transfection.

Hippocampal organotypic slice cultures were prepared from 7- to 9-d-old rats as previously described (11). CaMKIIα KO hippocampal organotypic slice cultures were prepared from 7- to 9-d-old mice. The CaMKIIα knockout mice were kindly donated from Bayer Lab. Transfections were carried out 48 h after slice preparation. Briefly, 50 μg DNA was coated on 1-μm-diameter gold particles in 0.5 mM spermidine, precipitated with 0.1 mM CaCl_2_, and washed four times in pure ethanol. The gold particles were coated onto PVC tubing, dried using ultrapure N_2_ gas, and stored at 4 °C in desiccant. DNA-coated gold particles were delivered with a Helios Gene Gun (BioRad). Slices were maintained at 34 °C with media changes every 2 d.

### Electrophysiological Recording.

All electrophysiological recordings were carried out on an upright Olympus BX51WI microscope and collected using a Multiclamp 700B amplifier (Molecular Devices). During recording, slices were maintained in ACSF (in mM): 125 NaCl, 2.5 KCl, 1.25 NaH_2_PO_4_, 25 NaHCO_3_, 11 glucose saturated with 95% O_2_; 5% CO_2_). For acute slice recording (Fig. 4), the ACSF contained 1 MgSO_4_, 2 CaCl_2_. For slice culture recording, the ACSF contained 4 MgSO_4_, 4 CaCl_2_, in order to dampen spontaneous activity. Transfected cells were identified visually using fluorescence and recorded simultaneously with a neighboring control cell. All recordings were carried out at 20 to 25 °C and used glass patch electrodes filled with an intracellular solution (in mM): 135 CsMeSO_3_, 10 HEPES, 8 NaCl, 0.3 EGTA, 4 Mg-ATP, 0.3 Na-GTP, 5 QX-314, and 0.1 spermine. Synaptic currents were elicited by stimulation of the Schaffer collaterals with a bipolar electrode (Micro Probes) placed in stratum radiatum. AMPAR- and NMDAR-mediated responses were collected in the presence of 100 µM picrotoxin to block inhibition. Then, 5 μM 2-chloroadenosine was used to suppress epileptic activity in slice culture. AMPAR EPSCs were recorded at −70 mV, and the amplitude was determined by measuring the peak of this response. NMDAR EPSCs were obtained at +40 mV and measured at 100 ms. Series resistances typically ranged from 10 to 20 MΩ; a cell pair was discarded if the series resistance of either cell increased to >30 MΩ. Statistical difference was determined using a two-tailed paired *t* test.

All LTP experiments (Fig. 4) were carried out in acute slices. LTP was induced via a pairing protocol of 40 Hz stimulation for 15 s at a holding potential of 0 mV, after recording a 3 to 5 min baseline, but not more than 6 min after breaking into the cell. Simultaneous dual whole-cell recordings were made in a transfected CA1 pyramidal cell and a neighboring WT cell. In some cases, one of the paired cells was lost during the experiment, then the recordings were considered until that point. In cases where one cell was lost, the recording was continued for the remaining cell was considered for the averages.

### Protein Expression and Purification.

WT and D135N mutant of CaMKIIα holoenzymes, referred to as Dead CaMKII, were expressed and purified according to the previous reported protocols (64, 67, 68). Briefly, variants of CaMKII with N-terminal His_6_-SUMO tag were coexpressed with λ phosphatase in *Escherichia coli* BL21-CodonPlus(DE3)-RIL cells (Agilent Technologies) in LB medium at 16 °C for 24 h. The recombinant CaMKII proteins were extracted by a high pressure homogenizer and purified by Ni^2+^-NTA Sepharose 6 Fast Flow resin (Cytiva) and Superose 6 26/600 (Cytiva) gel filtration chromatography. After the His_6_-SUMO tag was removed by Ulp1 protease, proteins were further purified by MonoQ anion exchange chromatography and Superose 6 10/300 gel filtration chromatography. The final column buffer as well as the storage buffer was 50 mM Tris pH 8.0, 200 mM NaCl, 10% glycerol, 5 mM DTT. All other proteins were expressed in *E. coli* BL21-CodonPlus(DE3)-RIL cells (Agilent Technologies) in LB medium at 16 °C overnight. Recombinant proteins were first purified using Ni^2+^-NTA Sepharose six Fast Flow affinity chromatography (Cytiva) for His_6_-tagged proteins or Glutathione Sepharose four Fast Flow affinity chromatography (Cytiva) for GST-tagged proteins and followed by a step of gel filtration chromatography using Superdex 200 26/600 or Superdex 75 26/600 column (Cytiva).

### GST Pull-Down Assay.

GST pull-down assay was carried out as previously described (64, 68). Briefly, HEK293T cells were transiently transfected with various GFP-CaMKII encoding plasmids using PEI, then harvested at 20 h posttransfection and lysed using the lysis buffer containing 50 mM Tris pH 7.5, 150 mM NaCl, 1% Triton X-100 and a cocktail of protease inhibitors (Merck). The mixture of lysate and GST-fused GluN2B (a.a. 1,259 to 1,310) was incubated at 4 °C for 30 min. After centrifugation at 16,873×*g* for 5 min, the supernatant was mixed with 40 μL of fresh Glutathione Sepharose resin and incubated for another 30 min. After extensive washing, the captured proteins were eluted by SDS-PAGE loading buffer by boiling, resolved by SDS-PAGE, and immunoblotted with GFP antibodies (Santa Cruz: sc-9996). Protein signals were visualized by an HRP-conjugated secondary antibody (Jackson ImmunoResearch: 715-035-150) and western HRP substrate (Sangon).

### CaMKII Kinase Activity Assay Using phos-tag SDS-PAGE.

CaMKII were expressed and purified according to the previous reported protocols (64). The cDNA sequence encoding Syntide-2 was cloned into a modified version of pET-32a with an N-terminal Trx-His_6_ tag (termed Trx-Syntide-2). Trx-Syntide-2 protein was expressed in *E. coli* BL21-CodonPlus(DE3)-RIL cells (Agilent Technologies) in LB medium at 16 °C. Recombinant proteins were purified using Ni^2+^-NTA Sepharose resin (GE Healthcare) and Superdex 75 26/600 gel filtration column (GE Healthcare). For CaMKII kinase activity assay using phos-tag SDS-PAGE, 15% acrylamide gel with 25 μM phos-tag acrylamide (AAL-107, Wako) and 250 μM MnCl_2_ were prepared for phos-tag SDS-PAGE. The experiments were carried out in the buffer containing 50 mM Tris pH 7.5, 150 mM NaCl, 1 mM CaCl_2_, 10 mM MgCl_2_, 0.5 mM ATP, 3 μM CaM, 1 μM CaMKII, and 100 μM Trx-Syntide-2. The reactions were quenched by 2× SDS loading buffer at different time points.

### Quantification and Statistical Analysis.

Statistical parameters including the definitions and exact values of n (e.g., number of experiments), distributions, and deviations are reported in the figures and corresponding figure legends. Statistical analysis was performed in GraphPad Prism.

## Supplementary Material

Appendix 01 (PDF)

## Data Availability

All study data are included in the article and/or *SI Appendix*.
