# Supplementary material for: CaMKII autophosphorylation is the only enzymatic event required for synaptic memory
Source: Proc Natl Acad Sci U S A. 2024 Jun 18;121(26):e2402783121. doi: 10.1073/pnas.2402783121 (PMC11214084; doi:10.1073/pnas.2402783121)
Supplement: Supplementary file 1 — Appendix 01 (PDF) [file pnas.2402783121.sapp.pdf]

PNAS

Supporting Information for

CaMKII autophosphorylation but not downstream kinase activity is required for synaptic memory

Xiumin Chen[1,2], Qixu Cai[3,4], Jing Zhou[5], Samuel J. Pleasure[5], Howard Schulman[6,7], Mingjie Zhang[3,8] and Roger A. Nicoll[2]*

[1]Department of Neurology and Institute of Neuroscience of Soochow University, Second Affiliated Hospital of Soochow University, Suzhou, 215004, China; [2] Department of Cellular and Molecular Pharmacology, University of California, San Francisco, San Francisco, CA 94158 [3]Division of Life Science, State Key Laboratory of Molecular Neuroscience, Hong Kong University of Science and Technology, Clear Water Bay, Kowloon, Hong Kong, China; [4]State Key Laboratory of Vaccines for Infectious Diseases, School of Public Heath, Xiamen University, Xiamen, Fujian 361102, China; [5]Department of Neurology, University of California, San Francisco, San Francisco, CA 94158; [6]Stanford University School of Medicine, Stanford, CA; [7]Panorama Research Institute, Sunnyvale, CA; [8]School of Life Sciences, Southern University of Science and Technology, Shenzhen, Guangdong 518055, China

*Address all correspondence to: Roger A. Nicoll

Department of Cellular and Molecular Pharmacology,

University of California at San Francisco

San Francisco, CA 94158

Email: roger.nicoll@ucsf.edu

Phone: (415) 450-0265

Figure S1

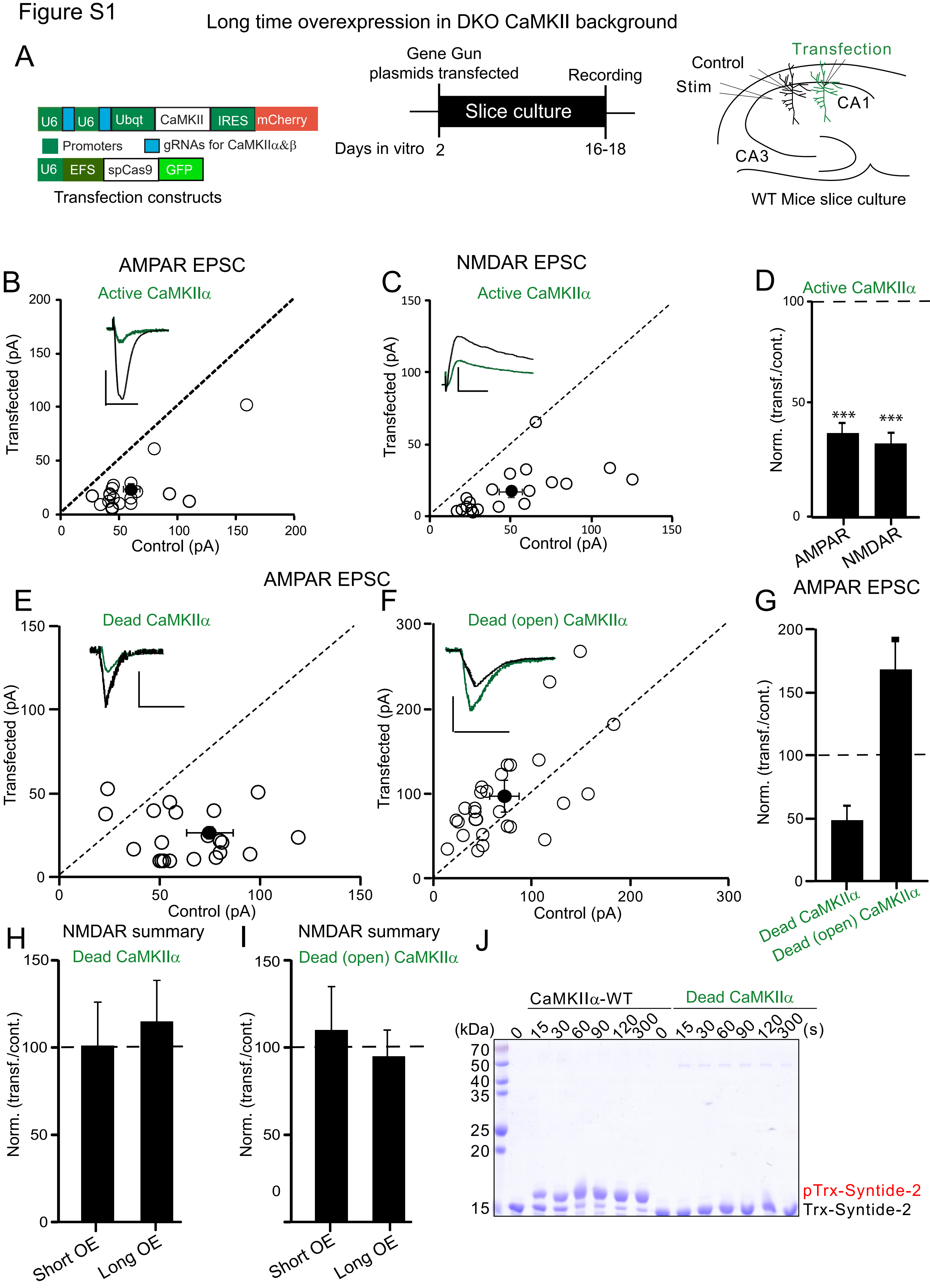

Long time overexpression in DKO CaMKII background
A
U6
U6
Ubqt
CaMKII
IRES
mCherry
Promoters
gRNAs for CaMKIIα&β
U6
EFS
spCas9
GFP
Transfection constructs
Gene Gun
plasmids transfected
Recording
Slice culture
Days in vitro 2
16-18
Control
Stim
Transfection
CA1
CA3
WT Mice slice culture
B
AMPAR EPSC
Active CaMKIIα
Transfected (pA)
Control (pA)
C
NMDAR EPSC
Active CaMKIIα
Transfected (pA)
Control (pA)
D
Active CaMKIIα
Norm. (transf./cont.)
AMPAR
NMDAR
AMPAR EPSC
E
Dead CaMKIIα
Transfected (pA)
Control (pA)
F
Dead (open) CaMKIIα
Transfected (pA)
Control (pA)
G
AMPAR EPSC
Norm. (transf./cont.)
Dead CaMKIIα
Dead (open) CaMKIIα
H
NMDAR summary
Dead CaMKIIα
Norm. (transf./cont.)
Short OE
Long OE
I
NMDAR summary
Dead (open) CaMKIIα
Norm. (transf./cont.)
Short OE
Long OE
J
CaMKIIα-WT
Dead CaMKIIα
(kDa)
0 15 30 60 90 120 300 0 15 30 60 90 120 300 (s)
70
50
40
35
25
20
15
pTrx-Syntide-2
Trx-Syntide-2

Figure S1. Prolonged Active CaMKIIα expression has a deleterious effect on synaptic transmission while Dead (open) CaMKII does not.

A Schematic diagram showing the transfection and electrophysiological approaches. **B** Scatterplots showing amplitudes of AMPAR EPSCs for single pairs (open circles) of control cells and overexpressing cells of Active CaMKIIα (CaMKIIα T286D-T305A-T306A) 14-16 days (long OE) (n = 14 pairs). Filled circle indicate mean ± SEM. (Control = 61.3 ± 7.2; Active CaMKIIα long OE = 23.7 ± 5.2 p < 0.0001). **C** Scatterplots showing amplitudes of NMDAR EPSCs for single pairs (open circles) of control cells and transfected cells of Active CaMKIIα 14-16 days (long OE) (n = 14 pairs). Filled circles indicate mean ± SEM. (Control = 50 ± 7.2; Active CaMKIIα 10 days = 16.9 ± 3.6, p < 0.001). **D** Bar graph of ratios normalized to control (%) summarizing the mean ± SEM of AMPAR and NMDAR EPSCs of values represented in **B** (38.5 ± 4.8, p < 0.0001) and **C** (32.6 ± 5, p < 0.0001). **E** and **F** Scatterplots showing amplitudes of AMPAR EPSCs for single pairs (open circles) of control cells and overexpressing cells of Dead CaMKII for 14 -16 days (long OE) (**E**, n = 25 pairs), and Dead (open) CaMKIIα for 14-16 days (long OE) (**F**, n = 20 pairs). Filled circle indicate mean ± SEM. (**E**, Control = 74.7 ± 11.7; Dead CaMKII long OE = 26.8 ± 3.6 p < 0.001; **F**, Control = 72.1 ± 14.8; Dead (open) CaMKII 14 days (long OE) = 98.2 ± 19 p < 0.01). **G** Bar graph of ratios normalized to control (%) summarizing the mean ± SEM of AMPAR EPSCs of **E** (49 ± 10, p < 0.001) and **F** (170 ± 21, p < 0.005). **H** Bar graph of ratios normalized to control (%) summarizing the mean ± SEM of NMDAR EPSCs of Dead (open) CaMKII 2-4 days (short OE) (101 ± 25, p =0.6) and 14-16 days (long OE) (110 ± 24, p =0.8). **I** Bar graph of ratios normalized to control (%) summarizing the mean ± SEM of NMDAR EPSCs of Dead (open) CaMKII expressed for 2-4 days (short OE) (115 ± 30, p =0.9) and for 14-6 days (long OE) (95 ± 15, p =0.7). **J** Kinase activity assay of WT CaMKIIα and the Dead CaMKIIα mutant with thioredoxin-fused Syntide-2 peptide (Trx-Syntide-2) as the substrate. Phosphorylation of Trx-Syntide-2 led to upshift of the Trx-Syntide-2 on the Phos-tag SDS-PAGE gel. The assay showed that Dead CaMKIIα is a catalytically null mutation of the enzyme. Raw amplitude data from dual cell recordings were analyzed using Wilcoxon signed rank test (p values indicated above). Normalized data were analyzed using a one-way ANOVA followed by the Brown-Forsythe test and Bartlett's test. Scale bars: 30 ms, 50 pA.

Figure S2

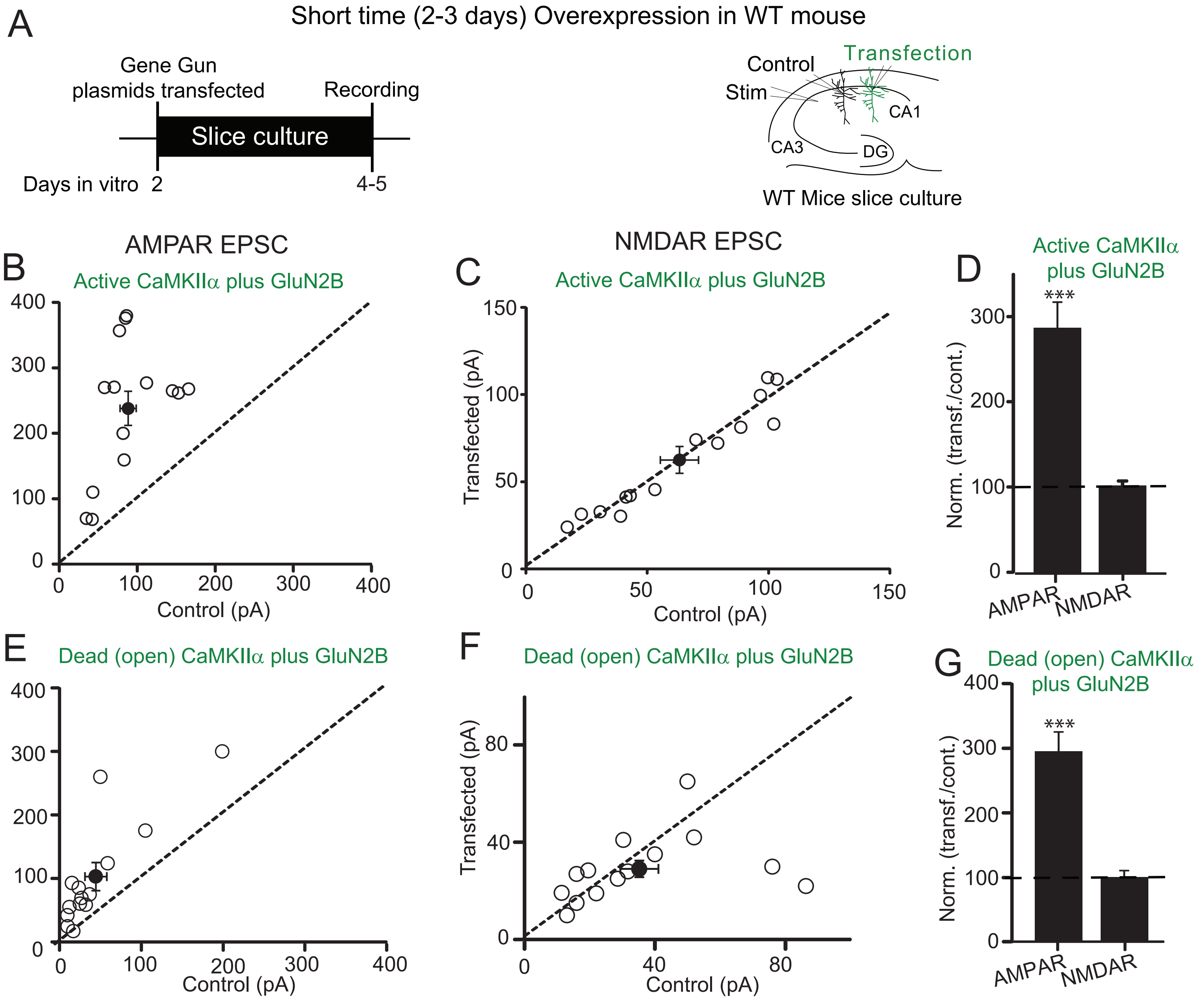
A
Short time (2-3 days) Overexpression in WT mouse
Gene Gun plasmids transfected
Recording
Slice culture
Days in vitro 2
4-5
Control
Transfection
Stim
CA1
CA3
DG
WT Mice slice culture
AMPAR EPSC
NMDAR EPSC
B
Active CaMKIIα plus GluN2B
Control (pA)
C
Active CaMKIIα plus GluN2B
Transfected (pA)
Control (pA)
D
Active CaMKIIα plus GluN2B
Norm. (transf./cont.)
***
AMPAR
NMDAR
E
Dead (open) CaMKIIα plus GluN2B
Control (pA)
F
Dead (open) CaMKIIα plus GluN2B
Transfected (pA)
Control (pA)
G
Dead (open) CaMKIIα plus GluN2B
Norm. (transf./cont.)
***
AMPAR
NMDAR

Figure S2. The action of Active CaMKII and Dead (open) CaMKII are not altered by co-expressing WT GluN2B

A Schematic diagram showing the transfection and electrophysiological approaches. Control represents the wild type, untransfected neurons. All experiments are from WT mice slice culture. **B** Scatterplots showing amplitudes of AMPAR EPSCs for single pairs (open circles) of controls cell and cells co-overexpressing Active CaMKII and GluN2B 2-3 days (short OE) ($n = 14$ pairs). Filled circle indicate mean ± SEM. (Control = 88 ± 10; Active CaMKII with GluN2B = 238 ± 26 $p < 0.0001$). **C** Scatterplots showing amplitudes of NMDAR EPSCs for single pairs (open circles) of control cells and co-transfected cells ($n = 14$ pairs). Filled circles indicate mean ± SEM. (Control= 63 ± 8; CA CaMKII with GluN2B = 62 ± 8, $p > 0.05$). **D** Bar graph of ratios normalized to control (%) summarizing the mean ± SEM of AMPAR and NMDAR EPSCs of values represented in B (287 ± 30, $p < 0.0001$) and C (102 ± 5, $p > 0.05$). **E** Scatterplots showing amplitudes of AMPAR EPSCs for single pairs (open circles) of control cells and cells co-overexpressing Dead (open) CaMKII and GluN2B 2-3 days (short OE) ($n = 13$ pairs). Filled circle indicate mean ± SEM. (Control = 44 ± 13; Dead (open) CaMKII with GluN2B = 103 ± 22 $p < 0.0001$). **F** Scatterplots showing amplitudes of NMDAR EPSCs for single pairs (open circles) of control cells and co-transfected cells ($n = 13$ pairs). Filled circles indicate mean ± SEM. (Control= 35 ± 6; Dead (open) CaMKII with GluN2B = 30 ± 5, $p > 0.05$). **G** Bar graph of ratios normalized to control (%) summarizing the mean ± SEM of AMPAR and NMDAR EPSCs of values represented in B (295 ± 30, $p < 0.0001$) and C (101 ± 9, $p > 0.05$).
